# Social justice in education: how the function of selection in educational institutions predicts support for (non)egalitarian assessment practices

**DOI:** 10.3389/fpsyg.2015.00707

**Published:** 2015-06-04

**Authors:** Frédérique Autin, Anatolia Batruch, Fabrizio Butera

**Affiliations:** Laboratoire de Psychologie Sociale (UNILaPS), Institut de Psychologie, Faculté des Sciences Sociales et Politiques, Université de Lausanne, Lausanne, Switzerland

**Keywords:** educational institutions, institutional practices, normative assessment, formative assessment, selection, social inequalities, justice beliefs, meritocracy

## Abstract

Educational institutions are considered a keystone for the establishment of a meritocratic society. They supposedly serve two functions: an educational function that promotes learning for all, and a selection function that sorts individuals into different programs, and ultimately social positions, based on individual merit. We study how the function of selection relates to support for assessment practices known to harm vs. benefit lower status students, through the perceived justice principles underlying these practices. We study two assessment practices: normative assessment—focused on ranking and social comparison, known to hinder the success of lower status students—and formative assessment—focused on learning and improvement, known to benefit lower status students. Normative assessment is usually perceived as relying on an equity principle, with rewards being allocated based on merit and should thus appear as positively associated with the function of selection. Formative assessment is usually perceived as relying on corrective justice that aims to ensure equality of outcomes by considering students’ needs, which makes it less suitable for the function of selection. A questionnaire measuring these constructs was administered to university students. Results showed that believing that education is intended to select the best students positively predicts support for normative assessment, through increased perception of its reliance on equity, and negatively predicts support for formative assessment, through reduced perception of its ability to establish corrective justice. This study suggests that the belief in the function of selection as inherent to educational institutions can contribute to the reproduction of social inequalities by preventing change from assessment practices known to disadvantage lower-status student, namely normative assessment, to more favorable practices, namely formative assessment, and by promoting matching beliefs in justice principles.

## Introduction

In most Western societies, educational institutions are perceived as an engine for social justice. By providing equal opportunities, education is believed to contribute to assign individuals to the academic and social positions that correspond to their aptitudes and motivation, regardless of their family’s wealth, background or social belonging. And yet, international surveys show that education fails to fulfill this role of “equalizer,” as pupils’ and students’ social background still strongly predicts their educational attainment (OECD, 2013a). These statistical trends show that the ideal of a meritocratic selection has yet to be reached. We propose that assigning to education the function of selecting the most deserving students could ironically participate in the reproduction of social inequalities. More precisely, in the present research we investigate how the belief that the function of educational institutions is to select students predicts the support for different kinds of assessment practices known to be more or less favorable to the disadvantaged, through corresponding beliefs in justice principles.

### The Two Functions of Educational Institutions

Throughout their modernization process, most industrial countries have regularly voiced concerns about establishing a fair society. One central question has been how to reconcile the commitment to equality and the existence of a stratified society. Indeed, as soon as equality of all humans became a fundamental value, the need to find a justifiable way of differentiating between individuals also emerged (Bisseret, 1974; Carson, 2007). The solution that was predominantly endorsed in the Western world was to ascribe social positions based on characteristics that seemed naturally distributed between individuals: abilities, ambition and efforts. In this context, educational institutions were given a crucial role. They became the place where these individual differences could be estimated and certified, relying on assessment methods rather than differences in social background. Thus, educational credentials, such as grades, certificates and diplomas, increasingly became a pass to access different social positions. However, several authors noted that, echoing the paradox between equality and differentiation, educational institutions fulfill two main functions, namely an educational and a selection function, whose articulation may need particular attention (Dornbusch et al., 1996; Darnon et al., 2009).

#### The Educational Function

First, mass education, which is a standard in most Western countries, offers equality of opportunity to all individuals, and is intended to develop every student’s potential. Educational institutions thus fulfill an *educational function* to the extent that they equip all students with knowledge, skills and capacities for learning. As stated in the Universal Declaration of Human Rights, “everyone has the right to education,” and educational institutions are supposed to safeguard this ideal. In practice, in Western societies, elementary education is compulsory and public schools offer a free access to all. Schools thus ensure that individuals master the basic knowledge and competences deemed necessary to take part in society (Parsons, 1959; Forquin, 1992; Dubet, 2004). This educational function is perceived as a way to foster social mobility (Bowen et al., 2005; Duru-Bellat, 2008): through the democratization of knowledge and increase of competence, education is expected to expand all individuals’ opportunities and warrant that no talent is wasted.

#### The Selection Function

Beside teaching skills and knowledge, education also serves a *function of selection*. Compulsory education makes opportunities available to all at first, but then individuals are trained for different social positions. Indeed, in most OECD countries, educational systems are divided into different types of programs, some being more vocational and others more academic. Even though the age at which students are sorted into different career tracks varies across countries, all educational systems carry out a more or less systematic and explicit selection (OECD, 2013b). At each successive tracking, only a fraction of the population moves to the most valued steps. Ultimately, only about 30% of adults have access to higher education (OECD, 2013c). It is important to note that attributing such a role of filter to educational institutions concords with the meritocratic ideal (Young, 1958). Indeed, it is now accepted that social positions should no longer be inherited but reflect individual merit. In education, merit is mostly defined as ability and motivation, qualities viewed in Western cultural models as intrinsic to the individual (Plaut and Markus, 2005) and educational institutions are perceived as a neutral place where individuals can express their inherent qualities. Then, to provide the most objective gage of individuals’ merit, educational systems rely on assessment procedures such as tests and exams that have become a basis for the selection of the most deserving students (Carson, 2007).

#### Selection and Education

However, research has long shown that beyond the rhetoric about a meritocratic selection based on individuals’ potentials, the reality is that socio-economic status (SES) is still related to academic outcomes. Indeed, several international surveys pointed to the fact that, compared to their socio-economically advantaged counterparts, disadvantaged students are more likely to underperform, repeat grades, drop out, and attain a lower level of education (OECD, 2010, 2013c). Eventually, disadvantaged individuals end up in lower status occupations and advantaged individuals in higher status positions, thus reproducing the social hierarchy existing prior to undergoing the educational process (OECD, 2010). Some scholars have claimed that, in fact, the functioning of educational institutions itself plays an active role in perpetuating the social hierarchy (Bourdieu and Passeron, 1977; Yosso, 2002). In this article, we focus on the perceived functions of educational institutions and the assessment practices enacted in these institutions. We propose that believing that educational institutions should select the best individuals is associated with more support for assessment practices that favor high status students and hinder the success of lower status students, and with less support for an assessment method that has the potential to reduce status-based performance gaps. On the contrary, the belief in the education role of school systems should be associated with less support for forms of assessment that reinforce inequalities and more support for egalitarian assessment practices. We also investigate how the relationship between the two functions of education and assessment practices is partly explained by the perceived justice principles underlying these practices.

### Assessment Practices and Social Inequalities

Some scholars suggested that educational institutions transform social inequalities into seemingly natural scholastic inequalities (Bourdieu and Passeron, 1977; Yosso, 2002). Indeed, many educational practices are conducive to the unequal treatment of students with differing social backgrounds. One of the most pervasive of these practices is assessment.

#### Normative Assessment

The most common assessment method in Western educational institutions is, by far, normative assessment, i.e., a form of evaluation based on a quantifiable measure of performance (e.g., numerical grades, letters, percentages or value judgments) that allows comparison to a social standard defining success (Knight and Yorke, 2003). One of the main characteristics of normative assessment is thus to reduce performance to a single indicator that is easily interpretable, which facilitates ranking and social comparison (Thorndike, 1913; Rosenholtz and Simpson, 1984), and makes it particularly useful to perform the function of selection (Dornbusch et al., 1996). Beside these structural features, it is also important to review some functional effects in relation to who is selected when using normative assessment.

Some historical and sociological analyses have proposed that normative assessment through testing and competitive examinations is rooted in traditions, methods, conceptions of knowledge and standards that serve the dominant groups (Wilbrink, 1997; Delandshere, 2001; Leathwood, 2005; Carson, 2007). The rankings and competence certification produced by normative assessment would thus participate to maintain the pre-existing social order. These analyses are corroborated by empirical research that (a) documented the deleterious consequences of normative assessment for students, especially from lower status groups and (b) investigated how normative assessment lead agents of the educational institutions to reproduce status-based achievement gaps.

As for the first body of empirical research, several results showed that normative forms of evaluation have deleterious consequences for learners. One consequence is that grades—which are typically used to perform normative assessment—lead students to be motivated by the desire to outperform others and the fear to be outperformed (Butler, 1987; Pulfrey et al., 2011). Such performance goals are associated with negative consequences such as self-handicapping (e.g., procrastination; Urdan et al., 1998) and superficial learning strategies (Nolen, 1988; Elliot and Harackiewicz, 1996; Meece et al., 2006). Moreover, students who were led to adopt performance goals by instructions that emphasized high stake performance and ranking experienced distractive outcome concerns that hijacked their cognitive resources and disrupted their performance (Crouzevialle and Butera, 2013).

This deleterious dynamic of performance goals may especially impact lower status students (Kaplan and Maehr, 1999; Jagacinski et al., 2008). Nicholls (1979) proposed that competitive contexts emphasizing normative evaluation and the demonstration of relative ability produce inequalities in the motivation necessary to develop skills and perform well. Recently, Smeding et al. (2013) provided a compelling demonstration that assessment practices oriented toward performance-based ranking particularly harm the academic achievement of low SES students. They showed that regular normative assessment (i.e., a final exam) and assessment experimentally emphasizing outperforming others impaired the performance of low-SES students, who then performed worse than high-SES students. The social class achievement gap, however, disappeared when assessment was experimentally presented to students as a way to learn and improve. Similar results were found on the gender-based achievement gap in science (Souchal et al., 2013). This body of research suggests that normative assessment in its usual form leads students to focus on demonstrating their ability and outperforming others and contributes to the lower achievement of lower status students.

A second body of research took the perspective of the agents of the educational system who enact the assessment practices, and set out to question the extent to which normative assessment reflects individual merit. More precisely, several studies revealed that the knowledge of the students’ social background could bias their teachers’ evaluation (Ouazad, 2008; Mechtenberg, 2009; Burgess and Greaves, 2013; Hinnerich et al., 2014). In an experimental study, in particular, German teachers were asked to grade a set of essays (Sprietsma, 2013). In all conditions, the essays were the same but the origin of the pupil’s name was manipulated. Some teachers thought a given essay was written by a native while others thought it was produced by a pupil with a migrant background. The results showed that the essays received lower grades when migrant pupils supposedly wrote them compared to the condition in which native pupils supposedly wrote them. Similar results were found in India where teachers gave lower grades to exams supposedly produced by low castes pupils compared to high castes pupils (Hanna and Linden, 2009).

In summary, normative assessment practices were historically implemented partly to fulfill the function of selection by allowing an objective detection of the most deserving students, notwithstanding their background. However, a growing set of evidence suggests that these assessment practices may backlash and contribute to the social reproduction of inequalities. We have shown how normative assessment may trigger psychological processes, in both students and teachers, that result in hindering the academic success of lower status students.

#### Formative Assessment

A number of alternative assessment methods have been developed to foster the learning of all students instead of favoring an elite or already advantaged groups. Research in education has long suggested that classroom environments oriented toward learning are more efficient (Nicholls, 1979; O’Neill, 1988; Wang et al., 1990; Crahay, 2012). The practices supporting a learning-oriented climate include cooperative learning, explicit teaching, clear and adapted instruction, maximized learning time and—most relevant for the present contention—alternative forms of evaluation.

Among these alternative forms of evaluation is formative assessment (Black and Wiliam, 1998). It is conducted during the learning process and is specifically intended to be a tool for improvement. The assessment can be conducted by the teachers, the students themselves or their peer and is formative to the extent that it provides a specific and detailed feedback that can be used to adapt the teaching and learning activities to the students’ progress and difficulties. Formative assessment can take various forms but we refer here to qualitative feedbacks provided to students that target specific learning objectives and provide guidance on how to improve (Torrance and Pryor, 1998; Shute, 2008; Bennett, 2011). Formative feedbacks inform the students about the desired outcome, the quality of their work compared to that standard and ways to attain it (Sadler, 1989). This kind of assessment practices is in line with the educational function of educational institutions because it aims at promoting the skills and knowledge of all students. On the contrary, formative assessment can hardly fulfill a selection purpose, as it is does not allow social comparison and ranking.

A thorough review of the literature showed that formative assessment has a strong, reliable and general positive impact on students’ learning (Black and Wiliam, 1998). For example, giving formative feedbacks to students reduced their fear of negative outcomes for an upcoming task. This relationship was mediated by higher autonomous motivation (i.e., behavior driven by individual’s goals or interest; Pulfrey et al., 2011; see also Pulfrey et al., 2013). In another study, pupils who received formative comments on previous exercises expressed a higher interest in the task, were ready to work on more extra tasks, and performed at a higher level on the subsequent task than students who received traditional forms of assessment (i.e., grades or praise; Butler, 1987).

There are reasons to think that lower status students could be those who benefit the most from formative assessment. Lower status students experience a mismatch with the norms and culture promoted in educational institutions (Stephens et al., 2012). Formative assessment makes the rules more transparent, by clarifying the expectations and how to meet them, which could help lower status students to adjust to the educational requirement. Lower status students also often feel that they “don’t belong here” and doubt their ability to succeed or think that others doubt their ability. Such experience of disqualification is known to impair performance (Cohen and Garcia, 2008; Croizet and Millet, 2012). Formative assessment shifts the focus from the evaluation of one’s self-worth as a student to evaluation as a way to improve and learn, which could limit lower status students’ concerns and help them achieve. And indeed, research has shown that, for female students, being oriented toward mastery and learning led in the long run to greater belief that they are capable of understanding the class and doing the required work, and to more use of strategies to monitor and control their learning (Patrick et al., 1999). Two literature reviews also showed that practices oriented toward learning, such as formative assessment, are especially beneficial to lower status students (O’Neill, 1988; Bissonnette et al., 2005). Using formative assessment could thus be a tool to reduce achievement inequalities between different social groups.

When comparing the literatures on formative and normative assessment, one may wonder why the latter is still the mostly used form of evaluation. We argue that support for these two assessment methods relates to the two functions of education. Support for normative assessment would be connected to selection purposes while support for the formative assessment would be linked to educational purposes. At the beginning of this theoretical section we have pointed out that much of the research on assessment has been motivated by the cultural belief that educational institutions should be an engine for social justice; thus, it is now time to discuss the justice principles that might underlie normative and formative assessment in educational institutions. Indeed, normative and formative methods imply different ways of allocating educational rewards, and different ways of treating the students during the learning process. These different principles of justice would make them more or less suitable to perform the selection and the educational function.

### Justice in Educational Institutions

#### Normative Assessment and Equity

The function of selection relies on a meritocratic ideal, whereby individuals are guided toward the position that corresponds to their dispositions. Historically, testing and graded exams were developed by measurement experts and psychologists to provide quantitative tools to a society based on individual merit (Lemann, 1999; Carson, 2007). The meritocratic ideology implies that rewards are allocated equitably, based on individual motivation, talent and hard work (Son Hing et al., 2011). The equity-based principle of justice is highly prevalent in school contexts and in particular in grade allocation (Sabbagh et al., 2006). Investigating teachers’ practices, Resh (2009) showed that they report using mostly equitarian rules to fairly determine grades, considering the student’s ability, success and effort. Interestingly, students share the idea that grade distribution should be guided by an equity principle (Jasso and Resh, 2002; Sabbagh et al., 2004). Such a consensus is captured by Deutsch’s (1979) theoretical contention that a fundamental function of normative assessment is to lead students to believe in meritocratic competition and in the fact that equity is the best way to allocate rewards.

The perceived reliance of normative assessment on the equity principle would explain why this method seems highly relevant to enact the function of selection. Because it seems to allocate rewards based on student’s merit, normative assessment appears as the best tool to select the most deserving students. On the contrary, the educational function would discourage the idea that assessment should establish an equity principle of justice. This function implies that all individuals should increase their level of competence, which is incompatible with an allocation of rewards based on the students’ initial input. The discouragement of the equity principle by educational purposes should relate to a perception of normative assessment as being an inadequate method.

#### Formative Assessment and Equality and Need

By contrast, formative assessment was developed with a view to improving the learning of all students. Reducing the gap between individuals who are unequal at the beginning of the pedagogic action is central to the rationale for implementing formative practices. These are framed as tools to institute a principle of corrective justice that ensures equality (Perrenoud, 1995; Dubet and Duru-Bellat, 2004; Crahay, 2012). It should be noted that equality in this case is not defined as the exact same treatment of all individuals during the learning process but as the equality of outcomes at the end of the learning process, obtained by a differentiated treatment of individuals as a function of their needs. Formative assessment is thus grounded in two egalitarian principles of justice: equality and need. The need principle implies to give more resources to those who need more (Deutsch, 1975): level, pace, content and methods should be adjusted to meet the students’ needs (Hallinan, 1988; Sabbagh et al., 2006). Formative assessment precisely aims at enabling such adjustments (Black and Wiliam, 1998): by giving learning opportunities adapted to each student, formative practices ambition to erase the original disparities in competence. All students should attain a high level of competence, and this level should be unrelated to their initial amount of skills. Ultimately, equality of outcomes would be established.

The equality and need principles of justice established by formative assessment fit the educational function of schools stating that all individuals should attain a certain level of skills and knowledge. On the contrary, the corrective justice inherent to formative assessment makes it incompatible with the function of selection. The need principle implies to identify individual differences but with the purpose of reducing them rather than using them to rank and attribute credentials. The ultimate principle of equality of outcomes is undifferentiating and cannot lead to selection.

### Hypotheses and Overview

Previous research has shown that normative and formative assessments contribute to respectively accentuate and attenuate social inequalities. In order to understand the support for these two assessment methods, we investigate how it relates to the selection and educational functions of educational institutions and justice principles. Firstly, we hypothesize that believing in the function of selection should be positively associated to the support for normative assessment practices. We expect this relationship to be mediated by the perception that normative assessment follows an equitarian principle of justice. Secondly, the belief in the function of selection should be negatively associated to the support for formative assessment practices. This lower support should be mediated by the reduced perception that formative assessment allows to meet the students’ need and ensure equality of outcomes. Thirdly, the belief in the educational function of education should relate to more support for formative assessment, this being mediated by a higher perception of its reliance on the need and equality principles of justice. Fourthly, the endorsement of the educational function should be negatively associated with support for normative assessment, through a negative relationship with the equity principle. To test our hypotheses, we administered a questionnaire measuring beliefs in the selection and the educational function of educational institutions, support for the normative and the formative assessment and the extent to which each assessment method follows each of three principles of justice (i.e., equity, equality and need).

## Materials and Methods

### Participants

One hundred and forty nine students enrolled in political science at a French-speaking Swiss university took part to the study. They voluntarily completed the questionnaire at the end of a regular class. Nine participants were removed from the analyses because they did not fill most of the questionnaire (*N* = 2), were not native French speakers (*N* = 3) or always gave the same answer (*N* = 4). The final sample included 140 students (mean age = 22.13, SD = 2.56; 73 women, 66 men, 1 unspecified). All data were collected in accordance to the American Psychological Association’s ethical principles and analyzed anonymously. This research was conducted in compliance with the declaration of Helsinki.

### Material and Procedure

Participants were first asked to imagine that they were secondary school teachers and, to commit them to this role-play, they had to list their supposed daily activities as a teacher. Then they had to fill in, on seven-point scales, a questionnaire developed to measure the functions of the educational system^1^. Three items referred to its function of selection (e.g., “The role of the educational system should be to deliver the best diplomas to the best students,” see items SelSys1 to SelSys3 in **Table 1A**) and three referred to its educational function (e.g., “The role of the educational system should be to help the students to gain solid knowledge,” see items EduSys1 to EduSys3 in **Table 1A**). Participants were presented with similar items to assess their perception of the selection vs. educational function of teachers (e.g, “As a teacher, your role is to give academic rewards only to the best students,” see items SelTea1 to SelTea3 and EduTea1 to EduTea3 in **Table 1A**).

**Table 1:** Standardized factor loadings.

A	Select	EduSyst	EduTeach	
*You think that the role of the educational system should be to*				
SelSys1 Detect among students those who are the most able to pursue their curriculum	0.57			
SelSys2 Deliver diplomas as a function of every student’s academic level	0.77			
SelSys3 Deliver the best diplomas to the best students	0.79			
EduSys1 Make sure that students master their course content		0.74		
EduSys2 Ensure that students’ knowledge increases		0.75		
EudSys3 Help students to gain solid knowledge		0.79		
*As a teacher, your role is to*				
SelTea1 Detect the students who have the greatest chances to successfully pursue their curriculum	0.54			
SelTea2 Make sure that students receive a diploma that corresponds to their academic level	0.75			
SelTea3 Give academic rewards only to the best students	0.58			
EduTea1 Make sure that all students master your course content			0.78	
EduTea2 Allow all students to increase their knowledge			0.77	
EduTea3 Help all students to gain solid knowledge			0.83	

**B**	**EquitNorm**	**EqualNorm**	**NeedNorm**	**SuppNorm**

*If you were to use this method (normative assessment), you would feel like*				
Equi1 This method allows to reward your students depending on the quality of their work	0.83			
Equi2 This method values your students as a function of their merit	0.72			
Equi3 This method enables you to give the best outcomes to your most talented students	0.38			
Equa1 This method allows you to take all your students to the same level of attainment		0.62		
Equa2 This method makes sure that all your students understood the class and can succeed		0.94		
Equa3 This method fosters all students’ learning		Excl.		
Need1 This method rewards your students for their effort and progress, regardless of how well they performed			0.74	
Need2 This method allows you to help your students as a function of their needs			0.64	
Need3 This method values your students even if they struggle			0.77	
Supp1 As a teacher you would use this method				0.80
Supp2 You think it is a good assessment method				0.84
Supp3 Your think it is a reliable assessment method				0.69
Supp4 You think it is a precise assessment method				0.60

**C**	**EquitForm**	**Equal/NeedForm**	**SuppForm**	

*If you were to use this method (formative assessment), you would feel like*				
Equi1 This method allows to reward your students depending on the quality of their work	0.84			
Equi2 This method values your students as a function of their merit	0.80			
Equi3 This method enables you to give the best outcomes to your most talented students	0.53			
Equa1 This method allows you to take all your students to the same level of attainment		0.70		
Equa2 This method makes sure that all your students understood the class and can succeed		0.70		
Equa3 This method fosters all students learning		Excl.		
Need1 This method rewards your students for their effort and progress, regardless of how well they performed		0.76		
Need2 This method allows you to help your students as a function of their needs		0.71		
Need3 This method values your students even if they struggle		0.62		
Supp1 As a teacher you would use this method			0.91	
Supp2 You think it is a good assessment method			0.94	
Supp3 Your think it is a reliable assessment method			0.75	
Supp4 You think it is a precise assessment method			0.76	

The second part of the questionnaire started with an explanation of the normative assessment method illustrated with a graded test. Participants read that this method is based on grades that reflect the number of right and wrong answers. Normative assessment was presented as enabling teachers to estimate the students’ learning, judge their performance according to a norm defining success and relatively to their peers. Participants then evaluated this assessment method on seven-point scales. Nine items assessed the justice principles (three items for each principle). Participants rated the fit of the assessment method with the equity principle (e.g., “This method values your students as a function of their merit,” see items Equi1 to Equi3 in **Table 1B**), the equality principle (e.g., “This method allows you to take all your students to the same level of attainment,” see items Equa1 to Equa3 in **Table 1B**), and the need principle (e.g., “This method values your students even if they struggle,” see items Need1 to Need3 in **Table 1B**). Finally, four items estimated the overall support for the method. Participants were asked whether they would use this method and whether they think it is a good, reliable and accurate assessment tool (see items Supp1 to Supp4 in **Table 1B**).

In the third part of the questionnaire, the formative assessment method was described and an example of a test with comment-based feedbacks was presented. Participants read that formative assessment is based on formative comments. This method was presented as enabling teachers to estimate the students’ learning, judge their performance according to learning objectives and suggest ways to improve. Participants filled in the same items measuring the three justice principles and the overall support for the method. The order of the second and the third part of the questionnaire was counterbalanced.

## Results

Relations between the perceived function of education, the justice principles followed by assessment methods and the support for these methods were estimated using structural equation modeling (SEM) analyses performed with the Lavaan package in R (Rosseel, 2012). First, confirmatory factor analyses (CFA) were used to identify the best-fitting measurement model. Then SEM examined the relationships among the latent variables and tested the specific hypotheses. The measurement model was identified by fixing the non-standardized factor loading of one of the indicators per latent variable to one. Our data being non-normal and incomplete, we used the Robust Maximum Likelihood (MLR) estimation method (Yuan and Bentler, 2000). The MLR estimator produces maximum likelihood parameter estimates with standard errors and χ^2^ test statistics that are robust to non-normality and missing data. Model fit was estimated by a number of convergent indices: the robust Yuan-Bentler scaled chi-square test, standardized root mean square residual (SRMR), the root-mean-square error of approximation (RMSEA) and the comparative fit index (CFI). Well-fitting model is suggested by a SRMR value below 0.08, a RMSEA close to 0.06 or below and a CFI value over 0.90 (Bentler, 1990; Hu and Bentler, 1999).

### Measurement Model

The sample size did not allow testing a model including all our variables. Our hypotheses imply that we investigate the relationship between the perceived functions of education and both the perception of the normative assessment, and the perception of the formative assessment. Consequently, we conducted separate analyses on the functions of education, the perception of normative assessment and the perception of formative assessment.

#### Functions of Education

The expected four-factor model, consisting of the selection and educational function of the educational system and the selection and educational function of teachers, showed a covariance matrix that was not positive definite. Inspection of the data suggested that this was caused by an overlap between two latent variables: the function of selection of the educational system and the function of selection of teachers (*r* = 1.12). Considering the similarity between the two sets of items, the two sets were integrated in a single variable referring to the function of selection of education. The reduced three-factor model showed a marginal fit Y-B χ^2^ (51, *N* = 140) = 106.96, *p* < 0.001, SRMR = 0.06; CFI = 0.90; RMSEA = 0.09 (90% CI of 0.07, 0.11, pclose = 0.003). Examination of modification indexes (MI) revealed correlation among error terms associated with two pairs of items: SelSys1 and SelTea1 (MI = 36.80), EduSys1 and EduTea3 (MI = 15.71). Such covariance can be explained by the substantial content overlap among the items. The correlation between the two pairs of error terms were added to the model one at a time, which significantly improved the fit (i.e., significant Satorra-Bentler-Scaled-χ^2^-difference-test; Δ SBS-χ^2^ = 14.69, *p* < 0.001 and Δ SBS-χ^2^ = 18.30, *p* < 0.001; Satorra and Bentler, 2001). The re-specified model showed a good fit Y-B χ^2^ (49, *N* = 140) = 58.97, *p* = 0.16, SRMR = 0.05; CFI = 0.98; RMSEA = 0.04 (90% CI of 0.000, 0.07, pclose = 0.69). The factor loadings, presented in **Table 1A**, were all significant (all *p*s < 0.001).

#### Normative Assessment

We hypothesized four latent variables, referring to the three principles of justice and the support for the assessment method. The four-factor model showed a moderate fit Y-B χ^2^ (59, *N* = 140) = 125, *p* < 0.001, SRMR = 0.07; CFI = 0.91; RMSEA = 0.09 (90% CI of 0.07, 0.11, pclose = 0.002). We inspected MI to assess whether the fit could be improved. The values indicated residual covariance of the item Equa3 with several other items. Given the multiple covariance, we decided to remove it, which improved the fit (BIC of 5768.75 compared to a BIC of 6245.16 for the original model, Raftery, 1995). Modification indices also indicated that the fit could be improved by allowing the errors of item Supp3 and Supp4 to correlate (MI = 20.57) as well as the errors of items Supp1 et Supp2 (MI = 18.39). These items refer to the same dimension of support. Allowing the residuals of these two pairs of items to be correlated further improved the fit (Δ SBS-χ^2^ = 10.81, *p* < 0.002 and Δ SBS-*χ*^2^ = 6.08, *p* < 0.02). The final re-specified model showed an excellent fit Y-B χ^2^ (46, *N* = 140) = 46.09, *p* = 0.47, SRMR = 0.05; CFI = 1; RMSEA = 0.004 (90% CI of 0.000, 0.06, pclose = 0.91). As shown in **Table 1B**, all indicators strongly loaded on the factors (all *p*s < 0.001).

#### Formative Assessement

We tested the four-factor model (i.e., three principles of justice and the support for the assessment method) and obtained an acceptable fit Y-B χ^2^ (59, *N* = 140) = 106.51, *p* < 0.001, SRMR = 0.05; CFI = 0.94; RMSEA = 0.08 (90% CI of 0.06, 0.10, pclose = 0.03). Inspection of the data indicated a high correlation between the equality and the need principle of justice (*r* = 0.92) and multiple covariance for the item Equa3. Because of the theoretical closeness of these two principles of justice, we decided to combine them into one latent variable referring to a principle of corrective justice that did not include the item Equa3, successfully improving the fit of the model (Δ BIC = 374). Based on modification indices, we allowed the errors of item Supp3 and Supp4 (that belong to the same theoretical dimension of support) to correlate (MI = 14.17) and the errors of items Equa1 and Equa2 (that refer to the dimension of equality; MI = 7.47). These successive changes improved the fit (Δ SBS-χ^2^ = 6.64, *p* < 0.01 and Δ SBS-χ^2^ = 4.92, *p* < 0.05). The fit of the final re-specified model was good Y-B χ^2^ (49, *N* = 140) = 69.97, *p* = 0.03, SRMR = 0.05; CFI = 0.97; RMSEA = 0.06 (90% CI of 0.02, 0.08, pclose = 0.36). **Table 1C** shows the all-significant factor loadings (all *p*s < 0.001).

### Structural Models

#### Normative Assessment

**FIGURE 1:**
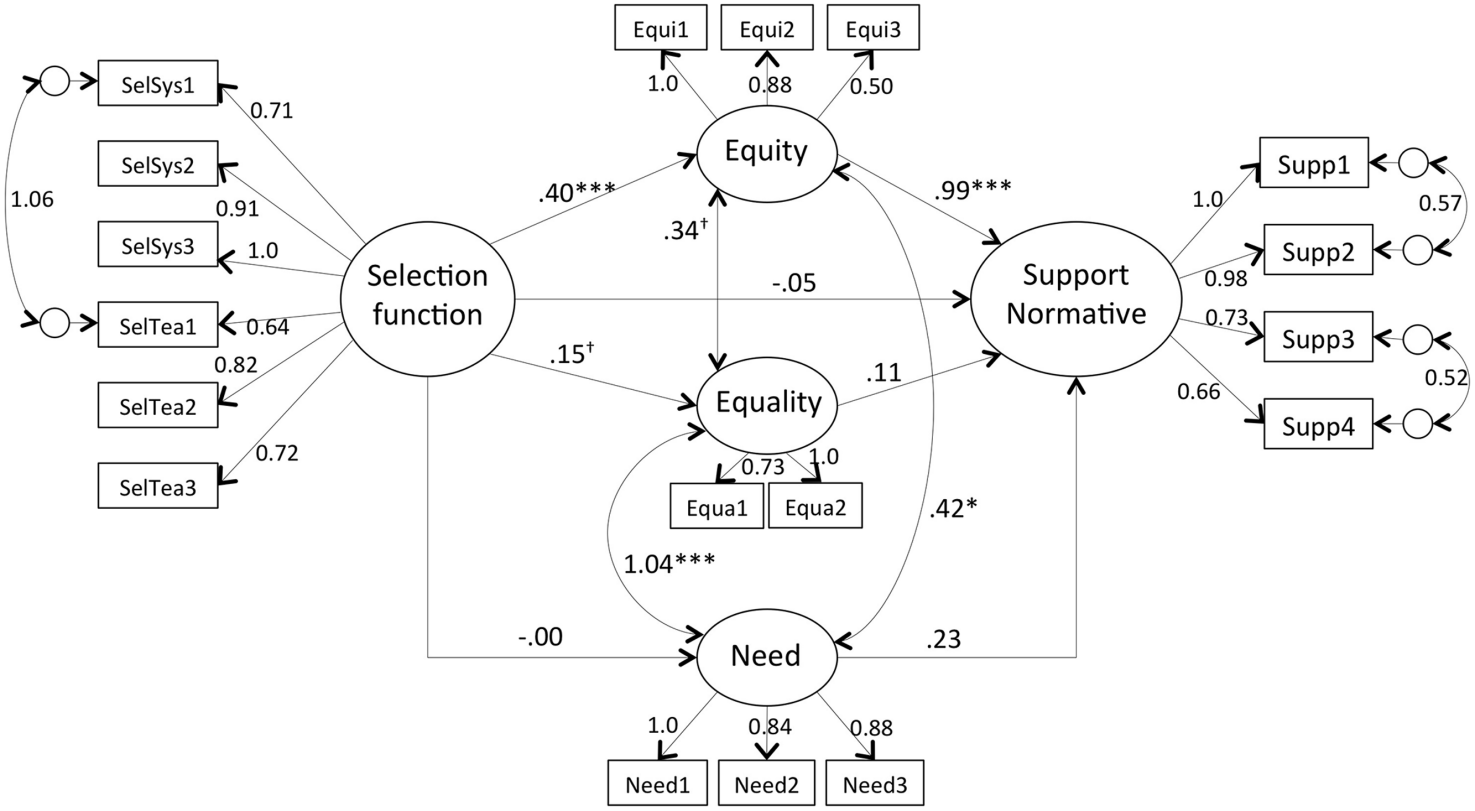
**Mediation model showing how the function of selection of education positively relates to the support for normative assessment via equity**. All values are unstandardized coefficients (†*p* < 0.10; ^∗^*p* < 0.05; ^∗∗∗^*p* < 0.001).

Descriptive statistics and zero-order correlations among variables are reported in **Table 2A**. The correlations are in the expected direction, except for the perceived educational function of school systems and teachers. We observed a ceiling effect and low variances, and therefore no correlation with other factors. This led us to exclude the two variables from the model, which prevented the test of Hypothesis 3. **Figure 1** shows the results of the structural equation model testing Hypothesis 1, stating that equity-based justice mediates the positive relation between the function of selection of education and the support for the normative assessment. The model fit the data well, Y-B χ^2^ (122, *N* = 140) = 139.76, *p* = 0.13, SRMR = 0.05; CFI = 0.98; RMSEA = 0.03 (90% CI of 0.000, 0.06, pclose = 0.89). In accordance with our hypothesis, the indirect path including equity-based justice was significant (*b* = 0.57, *z* = 3.66, *p* < 0.001) contrary to the indirect path including equality and need-based justice (respectively *b* = 0.02, *z* = 0.90, *p* = 0.37 and *b* = -0.001, *z* = -0.06, *p* = 0.96). Indicating a full mediation, the direct effect of the function of selection on the support for normative assessment was not significant (*b* = -0.08, *z* = -0.52, *p* = 0.60). These results indicate that thinking that education’s role is to select students relates to a positive evaluation of the normative assessment. This relation is mediated by beliefs that normative assessment allows to allocate rewards equitably.

**Table 2:** Descriptive statistics and zero-order correlations between the variables.

	*M*	SD	1	2	3	4	5	6	7
**(A)**
(1) Function of selection	3.95	1.33	–						
(2) Educational function system	6.34	0.80	0.14	–					
(3) Educational function teachers	6.31	0.80	0.10	0.73^∗∗∗^	–				
(4) Equity-based justice for the normative assessment	4.04	1.32	0.33^∗∗∗^	0.07	-0.05	–			
(5) Need based justice for the normative assessment	2.46	1.19	0.02	-0.05	-0.11	0.19^∗^	–		
(6) Equality based justice for the normative assessment	2.78	1.34	0.17†	0.01	-0.03	0.22^∗∗^	0.52^∗∗∗^	–	
(7) Support for normative assessment	3.76	1.47	0.30^∗∗∗^	0.07	0.001	0.69^∗∗∗^	0.37^∗∗∗^	0.35^∗∗∗^	–
**(B)**
(1) Function of selection	3.95	1.33	–						
(2) Educational function system	6.34	0.80	0.14	–					
(3) Educational function teachers	6.31	0.80	0.10	0.73^∗∗∗^	–				
(4) Equity-based justice for the formative assessment	4.15	1.43	-0.01	0.05	-0.02	–			
(5) Corrective (equality/need) justice for the formative assessment	4.99	1.21	-0.17^∗^	-0.03	0.06	0.60^∗∗∗^	–		
(6) Support for formative assessment	4.37	1.64	-0.19^∗^	-0.01	0.09	0.57^∗∗∗^	0.76^∗∗∗^	–	


*† *p* < 0.10; ^∗^*p* < 0.05; ^∗∗^*p* < 0.01; ^∗∗∗^*p* < 0.001*.

To test whether the links between variables was moderated by the order of presentation of the assessment methods, we performed multi-group SEM analyses. We first tested a model that introduced no equality constraints as a function of order. This unconstrained model was tested against a model in which all factor loadings were constrained to be equal across groups. The comparative fit of the two models indicated that the structure of the latent variables was similar in the two orders of presentation (Δ SBS-χ^2^ (13) = 14.45, n.s.). We then tested a model that constrained both factor loadings and all regression paths and covariances between latent variables to be equal across groups. Imposing equality constrains on the regression paths and covariances did not cause significant decrement in model fit (Δ SBS-χ^2^ (10) = 6.35, n.s.), suggesting that the structural relationships between the function of education, justice beliefs and support for the normative assessment was similar across order of presentation.

#### Formative Assessment

**FIGURE 2:**
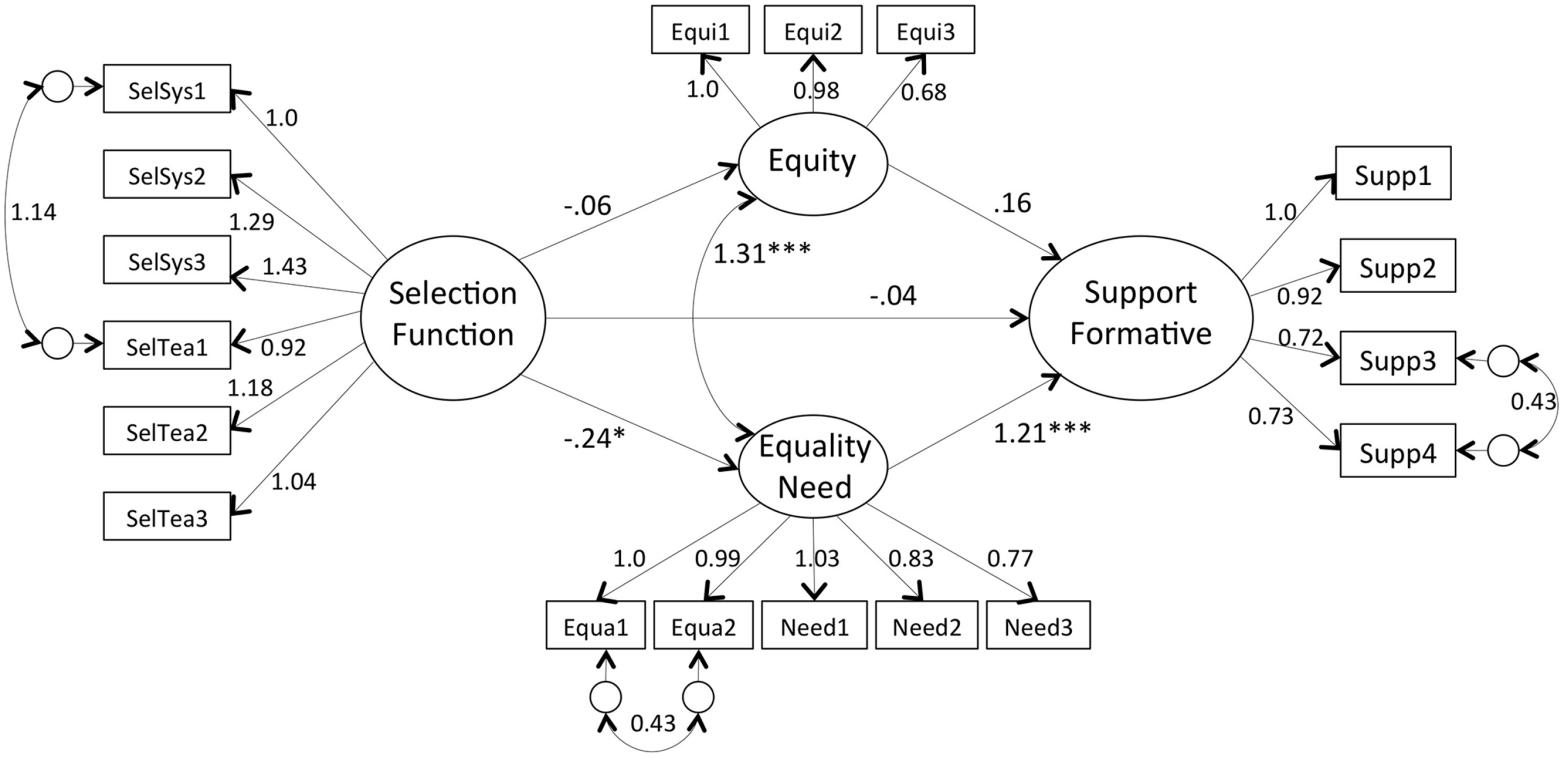
**Mediation model showing how the function of selection of education negatively relates to the support for formative assessment via corrective justice (equality and need)**. All values are unstandardized coefficients (^∗^*p* < 0.05; ^∗∗∗^*p* < 0.001).

We hypothesized that the support for formative assessment would be negatively related to the function of selection and that this relation would be mediated by the belief that this method follows an equality/need-based justice principle (Hypothesis 2). **Table 2B** shows the descriptive statistics and the zero-order associations between the variables that are consistent with our hypothesis. Again, the variables referring to the educational function did not correlate with any other variables and were excluded, which prevented the test of Hypothesis 4. Results of the structural equation model are shown in **Figure 2**. Despite the significant Y-B chi-square test [χ^2^ (126, *N* = 140) = 182, *p* = 0.001], other fit indices suggest a good fit, SRMR = 0.06; CFI = 0.95; RMSEA = 0.06 (90% CI of 0.04, 0.07, pclose = 0.25). The predicted indirect path including equality/need-based justice was marginally significant (*b* = -0.29, *z* = -1.94, *p* = 0.052) while the indirect path including equity-based justice was not significant (*b* = -0.01, *z* = -0.39, *p* = 0.69). The direct effect of the function of selection on the support for formative assessment was not significant (*b* = -0.04, *z* = -0.30, *p* = 0.77). These findings show that the beliefs stressing the role of selection of schools and teachers are negatively associated to the support for formative assessment, which is mediated by the reduced belief that formative assessment follows an equality/need-based justice principle.

Following the same logic as presented for the normative assessment, we tested the effect of the order of presentation of the assessment methods. The multi-group SEM analyses revealed that the structure of the latent variables was similar in the two orders of presentation [Δ SBS-χ^2^ (14) = 11.68, n.s.], as was the structural relationships between the function of education, justice beliefs and support for the formative assessment [Δ SBS-*χ*^2^ (6) = 10.34, n.s.].

## Discussion

The present study intended to uncover the beliefs about the functions of educational institutions, in particular the educational and selection functions, that may predict support for normative and formative assessment methods, since the type of assessment used has been found by previous research to either accentuate or attenuate social inequalities. To this effect, we used a questionnaire that allowed studying the relationships between the perceived function of the educational institution, the support for assessment practices, and the justice principles underlying these practices.

Our first hypothesis was that believing in the schools’ function of selection should be positively associated to the support for normative assessment practices, a relationship that should be mediated by the perception that normative assessment follows an equitarian principle of justice. In support to this hypothesis, we found that believing that the school’s role is to select the best students was positively associated to the support for normative assessment, a method known to be less favorable to lower status students. This relationship was indeed explained by the beliefs that such an assessment relies on an equity principle, one of the principles founding meritocracy (Son Hing et al., 2011). Our second hypothesis was that believing in the function of selection should be negatively associated to the support for formative assessment practices, a relationship that should be mediated by the reduced perception that formative assessment allows to meet the students’ needs and ensures equality of outcomes. The present results supported this hypothesis as well, and showed the expected negative relation between the function of selection and the support for formative assessment, a method favorable to lower status students. This relation was mediated by a reduced perception that formative comments rely on a corrective principle of justice aiming at bringing all the students to a similar level.

Unfortunately, we were unable to test our two hypotheses regarding the educational function of education, due to a ceiling effect and low variance in the variables referring to this construct. This problem is actually quite interesting, to the extent that it is likely to come from the fact that the educational function of school is widely recognized and endorsed, as it corresponds to the official discourse about the role of educational institutions (Darnon et al., 2009). Darnon et al. (2009) investigated the social value of mastery goals, the declared desire to learn and increase knowledge, in an academic context. These authors found that mastery goals are highly valued by students, both in terms of perceived desirability of these goals in the eyes of the teachers and in their perceived utility to succeed in the academic system. Interestingly, Darnon et al. (2009) also found that mastery goals perfectly fit the teachers’ discourse: when teachers were asked what goals they promoted in their class, their answers on mastery goals showed a ceiling effect and low variance. Mastery goals would be widely promoted by teachers precisely because they correspond to the educational function of education. These results and the similarity between the educational function of the educational system (to promote learning and increased knowledge, at the institutional level) and mastery goals (to strive for learning and increased knowledge, at the individual level) lead us to think that explicit questions about educational purposes are infused with social value issues, which will make it difficult for future research to study their link with other variables.

The question of how to partial social value out of the measure of the educational function should be addressed by future research (cf. Dompnier et al., 2013), but for the moment the present results on the function of selection represent an important contribution to the literature on the factors hindering and facilitating changes in the way educational agents perform scholastic and academic assessment. In the present research, we focused on two types of assessment practices: normative assessment, which is the most common method, and formative assessment, which is an alternative method. The cognitive and relational benefits of the latter for learners have been known for years (Black and Wiliam, 1998), and indeed in our own research participants even indicated stronger support for formative than for normative assessment [*t*(138) = -2.57, *p* = 0.01]. However, this is likely to be due to the high social desirability of focusing on education, as discussed in the previous paragraph, since the use of formative assessment in regular practices is still extremely limited (e.g., Black and Wiliam, 1998). A large body of literature has investigated why changes in assessment practices are difficult (Tierney, 2006). Many studies pointed to technical, political, and structural inhibiting factors, and to the role of teachers’ representation of teaching, assessment, learning and students (Hargreaves, 2005; Inbar-Lourie and Donitsa-Schmidt, 2009; Webb and Jones, 2009; Brown et al., 2011). Some proposed that the use of alternative assessment practices is hampered by institutional requirements, as well as the internalization of the institutional norms by the teachers who themselves succeeded in that system (Tabachnick et al., 1979; Hargreaves et al., 2002). Adding to this literature, our research provides empirical evidence that people’s endorsement of the function of selection of educational institutions relates to a greater support for the usual (i.e., normative) assessment practices and lower support for unusual (i.e., formative) assessment practices. Our results contribute to understand why, despite the growing evidence that normative assessment is detrimental for learners, change in practices is slow, by highlighting the role of the widespread idea that educational institutions are meant to select the best students. The difficulty to change assessment practices raises the issue of the benefit of normative and formative assessment for learners in general, but it may also have consequences for lower status students in particular. We have already mentioned the literature suggesting that normative assessment restrains the success of lower status students (e.g., Smeding et al., 2013) whereas formative assessment could benefit them (e.g., Bissonnette et al., 2005); consequently, the greater support for normative assessment and the lower support for formative assessment associated with the belief in the function of selection might result in perpetuating status-based achievement gaps. A possible extrapolation, and a suggestion for future research, is that the idea of a selection operated by educational institutions maintains social inequalities in the access to scholastic and professional opportunities.

Another contribution of the present research relates to justice beliefs. We found that the principle of equity, corresponding to a meritocratic allocation of rewards, positively relates to the support for an assessment method known to hinder the students from disadvantaged groups, namely normative assessment. On the contrary, the corrective justice, corresponding to an egalitarian principle, relates to more support for an assessment method that could benefit to lower status students, namely formative assessment. These findings are consistent with previous research showing that belief in meritocracy predicts support for organizational selection practices that sustain the status quo whereas egalitarian beliefs predict support for practices that challenge the status quo (Castilla and Benard, 2010; Son Hing et al., 2011; Zdaniuk and Bobocel, 2011). This body of research also demonstrated that meritocracy, besides being a justice principle, can serve as a hierarchy-legitimizing ideology. A possible extension of the present work could be to investigate whether the adherence to beliefs related to merit is a way to justify and legitimate the use of assessment practices known to disadvantage lower status students. Moreover, our results showed that such justification is positively associated to the extent to which one is convinced that educational institutions have the function of selecting students. A venue for future research could be to test the idea that bringing people to believe in the importance of selection at school leads to increased meritocratic beliefs that legitimize and maintain the current institutional functioning.

Several limitations of this work must be pointed out. First, the correlational nature of the data prevents from drawing any conclusion about the causal direction of the effects. We built our hypothesis on the idea that structural factors (i.e., functions of educational institutions) would affect beliefs about justice that in turn affect behavioral tendencies in the form of support for practices. Even though the observed relations are consistent with our hypothesis, we cannot claim that the function of selection leads to certain beliefs about justice and support for a specific assessment practice. Future research should manipulate the functions of education. We must note, however, that the problem related to the high social desirability of the educational function mentioned above might also curse such an experimental design, requiring subtle ways of inducing the selection and educational role of education. A second limitation relates to the measure of support for practices. We asked participants whether they would use each assessment method and whether they think they are good, reliable, and precise methods. We thus estimated behavioral intention and evaluation. Measuring actual behavior, for example by asking participants to assess a test, would allow investigating the enactment of these practices. Another limitation is the use of a student sample in this research. They were put in the position of a teacher by being asked to list their supposed daily activities as teachers and being reminded of their role in the framing of the questions. Research based on role-playing suggests that people are able to adapt their attitudes to a role they have been assigned to (Houston and Holmes, 1975; Covington and Omelich, 1979; Harari and Covington, 1981). A replication of this research with teachers would inform about potential differences between naïve conception of the educational institution and the conception of the agents of this system. Finally, our results apply to the Swiss context. In Switzerland, selection is explicit as children are systematically tracked at a young age (11–12 years old) and grades are supposedly the main criteria to make tracking decisions. Yet, we believe that the theoretical reasoning developed in this paper could be transposed to most educational systems in Western societies. Indeed, even if the function of selection of schools might be less explicit and practices may vary in different socio-cultural contexts, some form of selection is operated by most educational institutions (OECD, 2013b). For example, students can be grouped by ability, be granted/refused access to honor courses, have to pass competitive exams or selection might be operated at the admission stage (e.g., Sommet et al., 2013). Future research should investigate how various forms of assessment practices relate to justice principles and functions of education in contexts in which the function of selection is less explicit and systematic.

## Conclusion

Modern educational institutions have developed to become the warrant of a meritocratic society. Generalized access to education, and the implementation of supposedly objective measures of individuals’ motivations and abilities, were intended to lead to a fair society where desirable outcomes are distributed based on merit (Lemann, 1999; Carson, 2007). Adding to an abundant literature that demonstrated that this ideal is far from being achieved (Goldthorpe, 2003; Duru-Bellat, 2008; OECD, 2010; Walton et al., 2013), our results suggest that people’s beliefs in the importance of meritocratic selection relate to a willingness to sustain an institutional functioning, namely normative assessment, that is known to harm underprivileged students.

## Author Contributions

FA, AB, and FB conceived and designed the study. FA and AB collected the data and analyzed it under the supervision of FB. FA drafted the manuscript and AB and FB provided critical revisions. All authors approved the final version of the manuscript for submission.

## Conflict of Interest Statement

The authors declare that the research was conducted in the absence of any commercial or financial relationships that could be construed as a potential conflict of interest.

## Footnotes

1 Darnon, C., Dompnier, B., Buchs, C., Jury, M., and Butera, F. (in preparation). Selection and/or education: validation of a scale assessing the perception of the two functions of the educational system.
